# Preparing, Characterization and Anti-Biofilm Activity of Polymer Fibers Doped by Green Synthesized AgNPs

**DOI:** 10.3390/polym13040605

**Published:** 2021-02-17

**Authors:** Oksana Velgosova, Erika Mudra, Marek Vojtko

**Affiliations:** 1 Institute of Materials and Quality Engineering, Faculty of Materials, Metallurgy and Recycling, Technical University of Košice, Letna 9/A, 04200 Košice, Slovakia; 2 Division of Ceramic and Non-Metallic Systems, Institute of Materials Research, Slovak Academy of Sciences, Watsonova 47, 04001 Košice, Slovakia; emudra@saske.sk (E.M.); mvojtko@saske.sk (M.V.)

**Keywords:** Ag nanoparticles, green synthesis, poly(vinyl alcohol), polymer composite, electrospinning, microfibers

## Abstract

The aim of the work was to prepare polymer matrix composite (PMC) microfibers doped by green synthesized silver nanoparticles (AgNPs). The incorporation of AgNP into the polymer matrix can provide toxic properties to the polymer. Polyvinyl alcohol (PVA) was used as a matrix. AgNPs were synthesized by the green method, where the leaf extract of *Rosmarinus officinalis* (*R. officinalis*) was used as a reduction and capping agent. PVA-AgNPs composites were prepared in two ways: the ex situ method (pre-prepared globular AgNPs with a mean diameter of 20 nm were added into polymer matrix) and the in situ method (AgNPs were synthesized in the process of polymer composite preparation; in situ synthesized nanoparticles were a mix of different shapes with a mean diameter of ~100 nm). FTIR (Infrared spectroscopy with Fourier Transformation), UV–vis (Ultraviolet–visible spectroscopy), TEM (Transmission Electron Microscope), EDX (Energy-dispersive X-ray spectroscopy), and SEM (Scanning Electron Microscope) techniques were used for the analysis of nanoparticles and prepared PMCs. Thin layers and microfibers of in situ and ex situ PMCs were prepared. The presence of AgNPs clusters was evident in both PMC thin layers. After electrospinning, the chains of nanoparticles were observed inside the fibers. The distribution of nanoparticles was improved by increasing the AgNPs volume fraction (from 5 vol.% to 20 vol.%). Toxic and antibiofilm activity of AgNPs colloid, pure PVA, and PVA-AgNPs composites against the one-cell green algae *Parachlorella kessleri* (*P. kessleri*) was analyzed. AgNPs colloid, as well as PVA-AgNPs composites, showed good toxic and antibiofilm activity, and pure PVA shows no toxic/antibiofilm activity.

## 1. Introduction

Progress in science and technology constantly requires new materials that are characterized by special properties, or materials whose properties are enhanced by the addition of other substances that cause a significant change in the properties of the original material. Protection of the environment is also an important requirement in the process of new materials production. Therefore, great attention is currently paid to the production of materials by ecological, green methods. Nanomaterials and composite nanomaterials are currently at the forefront of interest. It is well known that nanomaterials have unique properties compared to bulk material. Nanosilver occupies an exceptional place among nanomaterials mainly due to its unusual optical, electrical, catalytic, and antibacterial properties [1,2].

There are many possibilities for the synthesis of silver nanoparticles (physical, chemical, and biological/green methods) [3]. All methods have certain advantages and disadvantages. Physical methods are generally more expensive, often require complex and energy-intensive equipment, and obtained particles are characterized by a wide size and shape range. Chemical methods are not as economically demanding, and there is not a problem to set the production conditions so that the prepared nanoparticles will have the required size. However, chemical methods often use environmentally unacceptable chemical compounds. On the other side, biological methods [4] have several advantages. They are relatively cheap and simple because they do not require complex technical equipment, and they are environmentally friendly because they use natural substances to reduce and stabilize metal nanoparticles. Algae [5], leaves [6], fruits [7], other parts of plants as peels [8], pomace [9] or extracts of plants or parts thereof, yeasts, fungi, and other biological material may be used in nanoparticle reduction [10]. The size and shape of nanoparticles prepared by green methods can be easily controlled by adjusting the conditions of the preparation such as temperature, pH of solutions, concentration, etc. Another advantage of green methods is that there is no need to add stabilizing agents or solvents to prevent aggregation of the prepared nanoparticles because plants contain a lot of biological agents and bioactive substances (antioxidants, polyphenols, polysaccharides, proteins, etc.), and functional groups and organic compounds can provide metal-reducing, capping/stabilizing effects.

The result of green synthesis is usually a colloidal solution of silver nanoparticles. The use of silver nanoparticles (AgNPs) in the aerosols or colloidal solutions for disinfection is well known, but the embedding of nanoparticles into the polymer matrix could facilitate the handling of AgNPs [11,12]. Moreover, polymers are promising materials due to their catalytic activity, thermo-sensitivity, and biocompatibility. The combination of silver nanoparticles with the polymer matrix can greatly improve the polymer properties (antimicrobial or smart materials can respond to stimuli such as temperature, pH, and electric or magnetic fields) and thereby expand their possibilities of application. Such smart polymers, whose original properties are improved, are at the forefront of scientific interest [13]. The incorporation of AgNP can provide polyvinyl alcohol (PVA) toxic properties that can be used in various industries, in this respect the most frequently mentioned industry is medicine. Other applications of such modified materials or their coatings can be very interesting, for example in areas where equipment or components are in constant contact with water (biofilm prevention).

A wide variety of polymers can be used as the matrix such as PVA [14,15], polyacrylic acid (PAA) [16], polyamide (PA) [17], polyallyalcohol [18], etc. There are also many possibilities for the polymer matrix composite (PMC) production (aqueous solution reduction [19], plasma polymerization [18], evaporation of polymers and metals, photo-irradiation, etc.). The solventless in situ reduction method of AgNPs in the PA (polyamide) matrix was described by Paridaa, D. et al. [17]. Applying this method, the authors successfully prepared PMC with uniform distribution of AgNPs. The prepared composite also displayed excellent antimicrobial activity. Quintero-Quiroz, C. et al. [20] describe the incorporation of AgNPs into a silicone elastomer. The prepared composite showed good antimicrobial activity against *Staphylococcus aureus* and MRSA (methicillin-resistant *S. aureus*). Furthermore, the AgNPs doped polymer exhibited good ultimate tensile strength, tensile rupture strength, and tensile elasticity modulus.

Despite the fact that many authors address this issue, preparation of PMC with good particle distribution, stability, and the required properties still remains a major challenge for scientists. The choice of a polymer depends on the intended use of the PMC [21].

The aim of this work was to prepare a PMC (PVA-AgNPs composite) with embedded green synthesized AgNPs. The benefits of green synthesis are indisputable. Therefore, in order to reduce production costs and simplify the synthesis process, the task was to prepare AgNPs by the biological method. *Rosmarinus officinalis* leaf extract was used for silver nanoparticles reduction. The composition of leaves extract before and after AgNPs synthesis, presence, shape, and size of synthesized AgNPs were analyzed by FTIR (Infrared spectroscopy with Fourier Transformation), UV–vis (Ultraviolet–visible spectroscopy), TEM (Transmission Electron Microscope), EDX (Energy-dispersive X-ray spectroscopy), and SEM (Scanning Electron Microscope) techniques.

Two methods of AgNPs incorporation, ex situ (nanoparticles were prepared in advance and added into the polymer matrix) and in situ (nanoparticles were synthesized directly in the polymer), were tested to determine which method is more appropriate, easier, and will ensure a more homogeneous distribution of nanoparticles. The thin layers (spin coater) and microfibers (electrospinning technology) of the PVA-AgNPs composite were prepared.

As a matrix, PVA—a water-soluble, biocompatible, non-toxic synthetic polymer determined for a variety of medical applications—was used. The presence, shape, size, and distribution of AgNPs in PMC were analyzed by the SEM technique.

To determine whether the incorporation of AgNPs improves the antibacterial properties of PVA, the toxicity and antibiofilm activity of AgNPs colloid, pure PVA, and PVA-AgNP composite were tested and analyzed.

## 2. Materials and Methods

### 2.1. Materials

Poly(vinyl alcohol) 98.0–98.8% product of ACROS Organics (Madrid, Spain) and silver nitrate A.G., a product of Penta Ltd. (Chrudim, Czech Republic), was purchased from FISHER Slovakia Ltd. (Bratislava, Slovakia). Fresh leaves of *R. officinalis* were collected on the outer part of the cadastral territory of the town of Kosice, Slovakia.

### 2.2. Preparing of AgNPs and PMCs

Polymer matrix composites (matrix—PVA; secondary phase—AgNPs (5 vol.%)) were prepared in two different ways:

1. Ex Situ prepared PVA-5AgNPs composite—to prepare the PVA-5AgNPs composite 8 wt.% solution of PVA and AgNPs were mixed together:
  (a) Preparing of 8 wt.% PVA solution—PVA powder and deionized water were mixed in a beaker, in a water bath at 70–80 °C for 2 h,
  (b) Synthesis of AgNPs—chemicals for experiment included the stock silver solution and leaves extract of *R. officinalis*:
    (i) the stock silver solution was prepared by dissolving of AgNO_3_ in deionized water (concentration of solution 50 mg/L Ag),
    (ii) the leaf extract was prepared using 10 g of fresh leaves of *R. officinalis*. Cleaned leaves were ground and mixed with 125 mL of deionized water, heated at 70 °C for 10 min, filtered, and centrifuged to remove solid residue; 100 mL of pure leaf extract was obtained,
    (iii) for the synthesis of AgNPs 20 mL of extract was added dropwise to 80 mL of stock silver solution heated to 70–80 °C. The pre-prepared 100 mL of AgNPs colloidal solution was thickened by centrifuge (3 mL of colloidal AgNPs was received).

Fifty milliliters of the 8 wt.% PVA solution and 3 mL of colloidal AgNPs were mixed by magnetic stirrer at 70–80 °C in a water bath, then put in an ultrasonic bath tank for better homogenization.

2. In Situ prepared PVA-5AgNPs composite-PVA powder, AgNO_3_ water solution, and *R. officinalis* leaf extract were mixed simultaneously in a beaker by magnetic stirrer at 80 °C in a water bath for 3 h. The amounts of the individual components were adjusted so that the concentration of PVA and Ag was the same as in the ex situ preparation process.

The ex situ and in situ PVA-5AgNPs composites were used for PMC thin layers and microfibers preparation. A spin coater was used for PMC thin layers preparation. Needle-less electrospinning technology (Nanospider) was used for PMC microfibers preparation. The applied voltage was 82 kV, and the distance between spinning and collector electrodes was 150 mm. All experiments were performed in triplicate.

### 2.3. Methods of Measurements

The presence of AgNPs was analyzed by a UNICAM UV–vis Spectrometer UV4 (Waltham, MA, USA). The size and morphology of the nanoparticles were studied by means of TEM (JEOL model JEM-2000FX, an accelerating voltage of 200 kV, Tokyo, Japan). ImageJ software (NIMH, Bethesda, MA, USA) was used for the analysis of AgNPs size distribution. Infrared spectroscopy with Fourier Transformation (FTIR) and an infrared microscope (NLIR, Farum, Denmark) were used to measure infrared spectra of the natural substance and AgNPs colloid. The morphology of PVA-AgNPs composite thin layers and fibers was observed by scanning electron microscopy SEM/FIB (SEM/FIB ZEISS-AURIGA Compact, Zeiss, Oberkochen, Germany). The phase composition of the samples was analyzed using energy dispersive X-ray analysis (EDX, ZEISS, Oberkochen, Germany).

### 2.4. Antibiofilm Activity

The toxicity of colloidal AgNPs and antibiofilm activity of pure PVA fibers and PVA-AgNPs composite fibers were evaluated using the slightly modified disk-diffusion method. Agar plates in Petri dishes were inoculated with *Parachlorella kessleri* (*P. kessleri*) algal cells. Sterile swabs wetted by 25 µL of prepared colloidal AgNPs were plated on agar plates for testing of colloidal AgNPs toxicity. The samples of PVA fibers and PVA-AgNPs composite fibers were plated directly on agar plates. The agar plates were then incubated at room temperature. The presence and size of the inhibition zone on the agar plates were checked after 14 days of growth.

## 3. Results and Discussion

### 3.1. Synthesis and Characterization of Pre-Prepared Silver Nanoparticles

**Figure 1:**
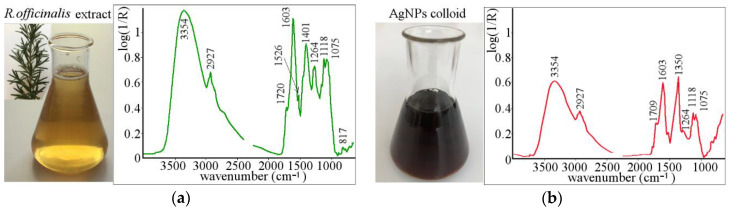
*Rosmarinus officinalis* leaves extracts and the FTIR analysis of the extract before AgNPs synthesis (**a**); the AgNPs colloidal solution and the FTIR analysis of AgNPs colloid (after nanoparticles synthesis) (**b**).

The left side of Figure 1a shows the *R. officinalis* leaf extract (light yellow, pure solution). Adding leaf extract into the stock solution (AgNO_3_ solution is clear) and subsequent synthesis of AgNPs caused the change in color of solution (it became dark brown), as shown on the right side of Figure 1b. The change in color is the first proof of AgNPs synthesis.

The FTIR analysis of extract, Figure 1a, showed the main functional groups of *R. officinalis*. *R. officinalis* is an evergreen aromatic shrub and contains a lot of natural antioxidants and essential oils [22,23]. It contains a number of phytochemicals including rosmarinic (caffeic acid ester), betulinic (pentacyclic triterpenoid), ursolic (pentacyclic triterpenoid), carnosic (diterpene, antioxidant, 1.5–2.5% in dried leaves), caffeic acids (consists of both phenolic and acrylic functional groups), and also camphor and carnosol (phenolic diterpene) [24,25]. The hydroxyl groups of phenolic compounds are considered as the main reducing and capping agents [22,26,27].

Figure 1b shows the FTIR analysis of the solution after AgNPs synthesis. The process of biosynthesis caused changes in intensities and positions of some peaks. In general, after AgNPs biosynthesis the decrease in all peak intensities was observed, and it can also be said that almost all main peaks remained at the same wavenumber positions. The FTIR spectra show broad hydrogen bonds at 3550–3200 cm^−1^, which are assigned to O–H stretching in phenols and alcohol compounds [28,29]. Hydroxyl groups and C–H stretches (alkyl groups present in most organic molecules) were observed at ~2927 cm^−1^ [30,31]. Significant shift with a simultaneous decrease in peak intensity was observed at 1401 cm^−1^ and 1720 cm^−1^; these peaks are attributed to free amino acid (CH_2_ bend) [23] and to the C–O–C stretching vibration band of the unsaturated ester carbonyl group [22,23], respectively. Blue shifting of the free amino acid peak from 1401 cm^−1^ to 1350 cm^−1^ and unsaturated ester carbonyl group from 1720 cm^−1^ to 1709 cm^−1^ with simultaneous decrease in their intensity indicates strong interaction of these groups with silver, or it can be considered as a contribution of said functional groups to the synthesis of AgNPs. Similar changes were observed by Morales et al. [32] in the synthesis of AgNPs using *Salvia hispanica* L. seeds. They attributed this to the interactions between individual functional groups and AgNPs, which led to the reduction of Ag^+^ ions to Ag^0^, through the oxidation of the hydroxyl groups. Based on the literature and our results we suppose that the depletion of the organic compounds (the decrease in peaks) can be considered as a more important factor for the process of AgNPs synthesis compared to the tiny shift (blue or red) of peaks.

The exact identification of the most important functional groups responsible for AgNPs formation and stabilization is not an easy task. In general, according to the decrease in the intensity of peaks, it can be concluded that all organic compounds take a part in AgNPs formation, and most of them act as reduction and also as stabilizing agents [26,29,33]. As leaf extract contains suitable reducing and stabilizing agents, the prepared AgNPs were stable (within 12 months), and no additional stabilizers needed to be added.

**Figure 2:**
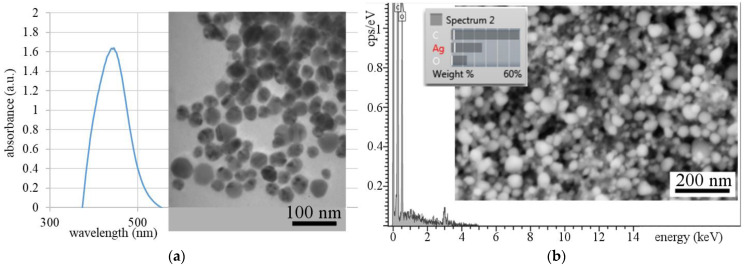
The UV–vis absorption spectrum of the AgNPs colloid, TEM micrograph of the AgNPs, and particle size histogram superimposed on the TEM image (**a**); SEM image and EDX analysis of the AgNPs (**b**).

AgNPs synthesis was also confirmed by UV–vis spectrophotometry (Figure 2a). AgNPs showed a strong peak at 446 nm. Based on the surface plasmon resonance (SPR) band shape (tall and slim) it is possible to assume that uniform nanoparticles with narrow size distribution were synthesized. This assumption was confirmed by a TEM micrograph (Figure 2a), where uniform nanoparticles with near-spherical morphology are evident. The size of the pre-prepared AgNPs was determined by image analysis from the TEM record. It is clear from the histogram that more than 86% of the AgNPs were up to 20 nm in size.

The SEM image and the EDX analysis of nanoparticles are shown in Figure 2b, where the near globular morphology of fine Ag nanoparticles is evident and the presence of silver was confirmed by EDX.

### 3.2. Characterization of Polymer Matrix Composite

It is well known that the distribution of nanoparticles in the matrix can be affected by a method of secondary phase incorporation. In general, the incorporation of metal nanoparticles into a polymeric matrix can be done using two different approaches: ex situ and in situ. To analyze the influence of incorporation methods on secondary phase distribution, and to select the best methods in terms of particle distribution, we applied both the ex situ incorporation method with pre-prepared silver nanoparticles and the in situ method where nanoparticles are formed in the PVA matrix in the process of composite preparation. The ex situ incorporation method is based on the physical entrapment of the metal nanoparticles into the polymer network [33,34]. In this case, a homogenous dispersion is difficult to obtain, but the advantage of this method is that the size and morphology of added particles are known in advance. To overcome the issue with the particle distribution, the in situ approach seems to be a better choice. Here, the metal particles are generated inside the polymer phase using a metal precursor, which is converted into the nanoparticles, but there is no guarantee of the size and shape of formed particles [33,35].

**Figure 3:**
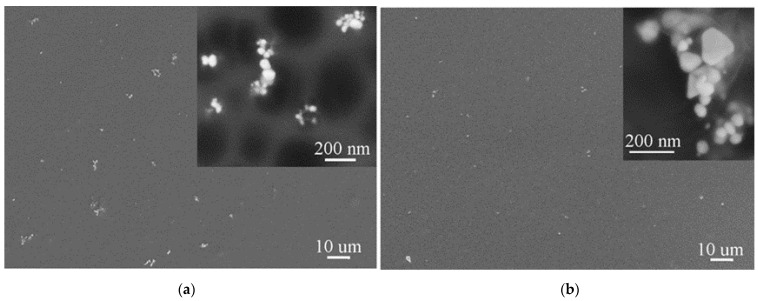
SEM images of ex situ (**a**) and in situ (**b**) PVA-5AgNPs thin layers with detailed view of AgNPs.

Thin films of both PMCs were prepared to determine the distribution of nanoparticles and analyze their size and shape. Since the shape and size of the silver nanoparticles used in the preparation of PMC by the ex situ method are known (spherical particles, diameter of 20 nm), we did not expect them to change. This assumption was confirmed by the SEM images, Figure 3a, of the thin layers of ex situ prepared PVA-5AgNPs composite. It is obvious that stirring the PVA and AgNPs mixture at elevated temperature and subsequent application of ultrasound did not ensure the appropriate homogenization. AgNPs formed clusters, the density of the PVA solution was probably too high, and the chosen incorporation condition (applied temperature and stirring conditions) cannot ensure desired dispersion of AgNPs.

**Figure 4:**
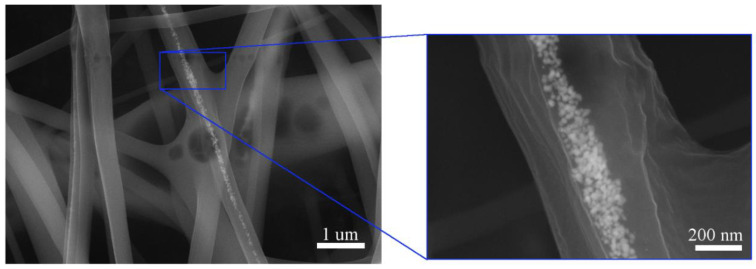
SEM images of ex situ PVA-5AgNPs fibers with detailed view of the AgNPs shape and distribution.

SEM images of ex situ PVA-5AgNPs microfibers prepared by electrospinning are shown in Figure 4. Since the SEM analysis, Figure 3a, showed the presence of clusters, we expected a similar result after the preparation of microfibers. The process of electrospinning did not help to improve the AgNPs distribution. After electrospinning, clusters presented in PMCs and formed chains of nanoparticles that were inside the fibers. Because of non-homogeneity in the AgNPs distribution, some of the microfibers contained nanoparticles and some did not, but it is evident that each cluster consisted of a number of nanosized AgNPs, Figure 4.

**Figure 5:**
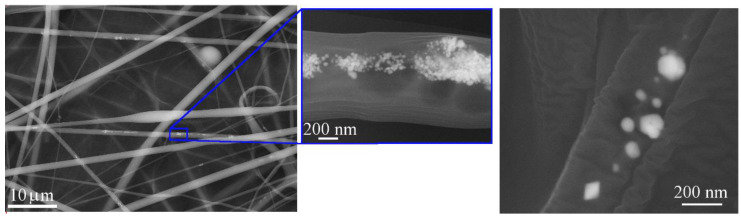
SEM images of in situ PVA-5AgNPs fibers with detailed view of the AgNPs shape and distribution.

Figure 3b shows the SEM images of thin layers of the in situ prepared PVA-5AgNPs composite. We assumed that the in situ method could affect the distribution of nanoparticles, as they should be formed directly in the PVA matrix. However, the SEM images showed that there was no difference in the AgNPs distribution compared to ex situ PMC, Figure 3a,b. Nanoparticles tended to form clusters despite the preparation methods. However, there was a difference in nanoparticles size and shape. The ex situ composite, Figure 3a, contained near globular particles with mean particle diameter of ~20 nm, but the in situ composite demonstrated non-homogeneity of the particles in terms of their size and shape. The near spherical (mean diameter of more than 40 nm) and irregular shapes such as hexagonal and triangular with varying particles size (in the range of 100–160 nm) were found, Figure 3b. Similar to ex situ composites, AgNPs were (after electrospinning) distributed in the chains inside the polymer fibers, Figure 5.

It was found that regardless of the method of AgNPs incorporation, neither the ex situ nor the in situ method influenced the distribution of nanoparticles. We assumed that longer and more intensive stirring at a higher temperature (70–80 °C), long-term exposure to ultrasound, and adjusting of PVA density could help to improve the AgNPs distribution.

The morphology and size of in situ formed nanoparticles were influenced by conditions of production. The formation of particles non-uniform in shape and size, which exceeded the nanoscale, can be considered as a disadvantage of the in situ method. Therefore, the next experiments (increasing of AgNPs content, toxic properties, and antibiofilm effect evaluation) were performed on a PMC prepared by the ex situ method. To analyze the influence of increasing AgNPs volume on distribution, the amount of incorporated nanoparticles was quadrupled. The experimental conditions for the preparation of a PMC with increased content of AgNPs were the same as in the previous cases (to ensure comparability of experiments).

**Figure 6:**
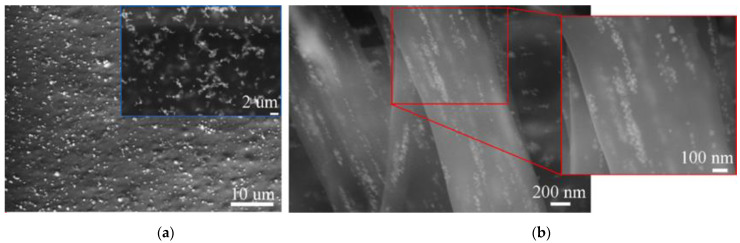
SEM images of ex situ PVA-20AgNPs (increased amount of nanoparticles) thin layers with detailed view on AgNPs (**a**); fibers with detailed view of the AgNPs distribution (**b**).

Figure 6a shows a thin layer of ex situ PMC with increased content of AgNPs (20 vol.%). It was clear that the nanoparticles, as in the low nanoparticle PMC, formed clusters in the PVA matrix, Figure 6a. The SEM image of PMC after electrospinning, Figure 6b, showed the presence of chains of the nanoparticles inside the polymer fibers. The increase in AgNPs content secured the presence of nanoparticles in all fibers.

### 3.3. Antibiofilm Effect

It is well known that silver is toxic to microorganisms. Notably, silver nanoparticles are often used in medicine in antibacterial and antifungal applications [19,36]. Numerous researchers studied the toxic effect of green synthesized AgNPs [15,22]. Most studies focused on testing the antibacterial (most commonly against *Escherichia coli*, *Pseudomonas aeruginosa* and *S. aureus* [36,37]) and antifungal (*Aspergillus oryzae*, *Candida albicans* [22]) properties of silver nanoparticles. In comparison, there were only a few studies that tested the toxicity of AgNPs against green one-cell algae, and so far none has addressed the effect of silver against biofilm formation. Biofilm-forming microorganisms have very specific properties. Once the conditions are in place for the cells to adhere to the surface, it is impossible to remove the biofilm. Therefore, we decided to test AgNPs and composites doped by AgNPs against biofilm formation.

**Figure 7:**
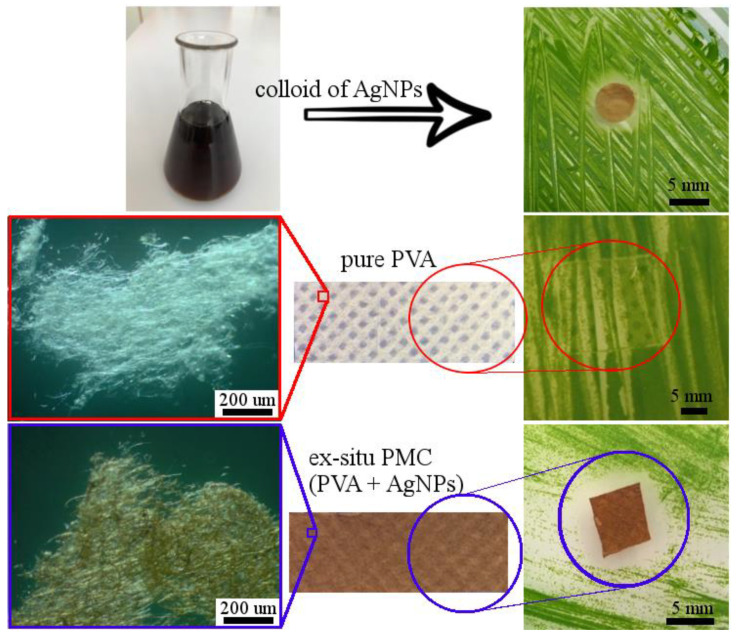
Toxicity test of colloidal AgNPs on one-cell green algae *P. kessleri*; toxic and antibiofilm effect of pure PVA and ex situ prepared PVA-AgNPs composites with a detailed view on fibers (in top-down order).

The toxicity of silver nanoparticles was verified on one-cell green algae *P. kessleri*. Figure 7 shows the comparison of the toxic effect of AgNPs colloid, the antibiofilm effect of pure PVA fibers, and ex situ prepared PVA-20AgNPs PMC.

It is obvious that colloid of green synthesized AgNPs (~20 nm in diameter) created a clear inhibition zone with a mean diameter of ~7 mm. The presence of such an evident inhibition zone confirmed the good toxic properties of green synthesized AgNPs.

To analyze the ability to prevent biofilm growth (antibiofilm effect) the fibers of pure PVA and ex situ PVA-20AgNPs composite were compared, Figure 7.

Samples for testing of polymers were taken from the nonwoven fabric prepared by the electrospinning method, Figure 7. A detailed view of the fibers of both samples (PVA and PVA-20AgNPs) is also shown in Figure 7. Samples were placed on algal inoculated agar. After 14 days, the samples were evaluated, and it was found that the pure PVA fibers did not show any toxic or antibiofilm effects. The green algae had overgrown the sample completely. A different situation occurred with the fibers of the PVA-20AgNPs composite. It is clear that the composite fibers not only prevented the formation of biofilm but also showed a significant inhibition zone (8–9 mm in diameter).

The improving/obtaining of antibacterial, toxic, or antibiofilm formation properties offers a wider range of uses for materials. Thin films and nonwovens of polymers doped with AgNPs could be used to protect surfaces in environments with high humidity, in the water industry, in medical applications, and also in food packaging.

## 4. Conclusions

Polymer matrix composites doped by AgNPs (PVA-AgNPs composites) were successfully prepared. Silver nanoparticles were successfully prepared by green synthesis, and an extract of *R. officinalis* leaves was used. Green synthesis has been used to reduce the cost and to simplify the PMC preparation process, as it is simple, fast, does not require complex equipment, and is environmentally friendly.

Two different methods of AgNPs incorporation, ex situ and in situ, in terms of efficiency and distribution of nanoparticles in PMC were applied. The thin layers and microfibers of ex situ (5 vol.% and 20 vol.% of AgNPs) and in situ (5 vol.% of AgNPs) PVA-AgNPs composites were prepared. Thin layers of PMC with a lower amount of AgNPs showed non-homogeneity in AgNPs distribution. The increase in the content of AgNPs (PVA-20AgNPs) caused an improvement in the distribution of nanoparticles, but a slight tendency to agglomeration was observed. The method of incorporation of AgNPs significantly affected the morphology and size of the AgNPs. From the results it is clear that the in situ process is not suitable in set production conditions (AgNPs of a different shape were observed, and most of the formed nanoparticles exceeded the nanoscale).

We suppose that for improving the AgNPs distribution it will be necessary to adjust the incorporation part of the process, and the density of the PVA solution can also play an important role. Our next goal is to improve the distribution of nanoparticles in the matrix and to achieve their even distribution not only inside the fibers but also on their surface.

The toxic properties and antibiofilm effect of the samples of AgNPs colloid, pure PVA fibers, and ex situ prepared PMCs were tested against the one-cell green algae *P. kessleri*. No inhibition zone around the pure PVA sample was created, and no antibiofilm effect was observed. However, the PMC fibers and AgNPs colloid showed good toxicity (clear inhibition zones were created) and antibiofilm effects.

## Data Availability

The data presented in this study are available on request from the corresponding author.
